# Menstrual hygiene management among adolescent schoolgirls in low- and middle-income countries: research priorities

**DOI:** 10.3402/gha.v9.33032

**Published:** 2016-12-08

**Authors:** Penelope A. Phillips-Howard, Bethany Caruso, Belen Torondel, Garazi Zulaika, Murat Sahin, Marni Sommer

**Affiliations:** 1 Department of Clinical Sciences, Liverpool School of Tropical Medicine, Liverpool, UK; 2 Department of Environmental Health, Rollins School of Public Health, Emory University, Atlanta, GA, USA; 3 Department of Disease Control, London School of Hygiene and Tropical Medicine, London, UK; 4 WASH Section, Programme Division, United Nations Children Fund, New York, NY, USA; 5 Department of Sociomedical Sciences, Columbia University Mailman School of Public Health, New York, NY, USA

**Keywords:** equity, adolescent sexual and reproductive health, menstruation, hygiene, school health

## Abstract

**Background:**

A lack of adequate guidance on menstrual management; water, disposal, and private changing facilities; and sanitary hygiene materials in low- and middle-income countries leaves schoolgirls with limited options for healthy personal hygiene during monthly menses. While a plethora of observational studies have described how menstrual hygiene management (MHM) barriers in school impact girls’ dignity, well-being, and engagement in school activities, studies have yet to confirm if inadequate information and facilities for MHM significantly affects quantifiable school and health outcomes influencing girls’ life chances. Evidence on these hard outcomes will take time to accrue; however, a current lack of standardized methods, tools, and research funding is hampering progress and must be addressed.

**Objectives:**

Compile research priorities for MHM and types of research methods that can be used.

**Results:**

In this article, we highlight the current knowledge gaps in school-aged girls’ MHM research, and identify opportunities for addressing the dearth of hard evidence limiting the ability of governments, donors, and other agencies to appropriately target resources. We outline a series of research priorities and methodologies that were drawn from an expert panel to address global priorities for MHM in schools for the next 10 years.

**Conclusions:**

A strong evidence base for different settings, standardized definitions regarding MHM outcomes, improved study designs and methodologies, and the creation of an MHM research consortia to focus attention on this neglected global issue.

## Introduction

Young persons aged 10–24 years represent a quarter of the world's population, with 90% living in low- and middle-income countries (LMICs); of these, 500 million are girls aged 10–19 years living in less-developed countries (1). Adolescence is a critical time of psychological and biological change, and there is strong recognition of the disproportionate sexual and reproductive health (SRH) harms placed on adolescent girls in LMICs (2–4). Evidence of a positive association between girls’ education, health, and economic potential has strengthened international resolve to improve educational opportunities for adolescent girls (5, 6). One challenge adolescent girls face in school in LMICs is managing their menstruation amid other pubertal changes (7).

Despite recent momentum and a codified definition of what menstrual hygiene management (MHM) necessitates (see Box 1), a lack of adequate guidance on MHM; poor quality and an inadequate supply of water, disposal facilities, and privacy for changing in many schools continue to leave girls with limited options for safe and proper personal hygiene (8–14). In addition, a lack of adequate sanitary hygiene products forces some girls to use unhygienic materials (11, 13, 15, 16), potentially increasing urogenital symptoms and infections (17). New but limited evidence suggests that this need leads adolescent girls to engage in transactional sex in order to buy menstrual products (18, 19). Moreover, inadequate social support, ongoing gender inequality, and social and hygiene taboos around menstruation in numerous countries leave girls experiencing shame, fear, and confusion when trying to cope with their menstrual flow (9–15, 20, 21). In addition to stripping girls of their self-esteem and sense of agency, growing evidence suggests that inequitable school environments negatively impact girls’ ability to succeed academically, limit their long-term economic potential, and significantly affect their SRH outcomes (5, 6, 11, 13, 14, 20–23). While there is acknowledgement that poor MHM is a neglected social issue preventing girls from managing their menstruation with safety, dignity, and privacy, research on its effect on girls’ lives remains limited (7, 24).

*Box 1*. Joint Monitoring Programme of WHO/UNICEF Definition of Adequate MHM (35)

Women and adolescent girls are using a clean menstrual management material to absorb or collect menstrual blood, that can be changed in privacy as often as necessary for the duration of a menstrual period, using soap and water for washing the body as required, and having access to facilities to dispose of used menstrual management materials.

In recognition of the need to improve the experience of MHM for girls in LMICs, researchers and practitioners met to outline a global vision for MHM in schools by 2024 and identified priority action areas to guide global, national, and local action (7). This article focuses on one of those priorities: the need for a strong cross-sectoral evidence base for MHM in schools for effective policies, resource allocation, and programming at scale to ameliorate MHM as a socially neglected issue. The lack of evidence continues to be a major impediment to progress, with a lack of data contextualizing the contribution good MHM can make to improving girls’ lives. This hampers the ability of governments, donors, and others to prioritize and appropriately target resources (7). Here, we outline the critical research topics that were identified as global necessities for MHM for the next 10 years and discuss the methodological implications to close the gaps in the existing MHM in schools’ evidence base.

## The need for a strong evidence base

MHM-related research in LMICs has primarily focused on describing the challenges and barriers associated with MHM among schoolgirls, in limited cultural and geographical settings, with early initiatives examining the effect of potential interventions (15, 25, 26). Given the dearth of information on MHM globally, the expected sensitivities around the topic, and the lack of standardized tools and methods, research has predominantly provided evidence from qualitative, participatory, and descriptive methods. While this has built a foundation for action to counter girls’ unmet MHM-related needs (9), a commensurate number of analytical studies have not been performed (27).

There are a number of priority research areas that have been identified as essential for shaping and promoting MHM in schools. Advancing the evidence on these topics will enable the global community to better understand the main negative effects of poor MHM on girls’ well-being, dignity, health, and schooling. This includes strengthening the body of knowledge around MHM's impact on school dropout, absence, and other measureable school indicators (such as stress, self-confidence, and self-efficacy). Efforts to specifically explore the impact of poor MHM on girls’ SRH are also needed to define its contribution toward sexual exposure (18, 19), and subsequent increased risk of schoolgirl pregnancies and sexually transmitted infections (23). Laboratory-based support is required given the limited predictability of girls’ (and women's) reported symptomatology (17, 23, 28). There is also a need to design good and innovative MHM interventions (individually or as a package) for different settings, to evaluate their impact on different outcomes and to measure their sustainability and cost-effectiveness.

The existing MHM evidence to date, which is comprehensive in terms of the barriers facing girls in school across many LMICs, thus provides little verification of critical outcomes affecting girls’ lives and has not fully addressed why MHM remains a neglected issue. While inequitable school environments have been shown to disadvantage girls and increase the risk of pregnancy and dropout (29, 30), evidence of the MHM-specific effects are unknown (27). Ascertainment of intermediate outcomes such as school absence (i.e. which may contribute to dropout or failure to achieve) has been mixed (14, 31), with no strong impact shown after provision of menstrual products (15, 32). No studies have been conducted on girls’ level of attainment in school (27). A sole focus on school misses exploration of important health effects on girls’ SRH and wider effects on girls’ life chances; while pilot studies have explored this (33), verification through larger trials is required. As well as the effect of transactional sex for menstrual products (18, 19), the potential for unintended pregnancy due to a lack of girls’ understanding between the menstrual cycle and fertility has been documented (34), but not studied further.

The topic areas shown in Table 1 are exemplars identified by researchers and practitioners as having critical gaps in knowledge around MHM for adolescent girls (7). Addressing these research topics in multiple sites is essential to understand the breadth and depth of MHM needs as well as to highlight cultural differences in MHM that are critical to intervention roll-out and adoption.

**Table 1:** Exemplar topics and research questions around MHM for adolescent girls

Topic area	Research question
Neglect to address MHM issues	Why does menstrual need continue to be socially neglected? What interventions are required to influence social norms across cultures and improve MHM worldwide?
Environmental infrastructure	Do WASH infrastructure improvements impact girls’ ability to attain equitable educational outcomes as boys (with or without a specific menstrual product intervention)? Do girls use the improved infrastructure provided for menstrual management? Do WASH improvements ameliorate girls’ MHM challenges in the school setting?
	What are cost-effective menstrual waste disposal systems? How can safe, hygienic, sustainable, and environmentally friendly disposal systems be developed?
Hygiene products	How can programs improve access to menstrual products, such as sanitary pads, other absorbents, or menstrual cups, and availability of underwear? Are certain MHM products only culturally acceptable in some countries? How does culture or religion effect uptake? Can acceptability and use be promoted globally?
	How can programs measure the benefits and risks of traditional hygiene materials (such as cloth) in LMIC and support safe practices?
	Can cluster randomized controlled trials define the cost-effectiveness of MHM products on hard outcome measures?
School-based programming	What MHM program delivery mechanisms effectively ensure provision for schoolgirls?
	What is the effectiveness of psychosocial support programs delivered through teachers, nurses, or counselors?
	Is MHM education in schools a global necessity regardless of measureable health or school outcomes?
Delivery channels	What modes of MHM service delivery best ensure girls in greatest need are served?
	What are the needs of girls with disabilities and what guidance is required to support them?
	What is the design of an effective evidence-based community- or school-delivery and support program for refugees, orphans, street kids, or girls not in school?
Girls’ health	What health impact would MHM products have on reproductive tract infections, vaginal discharge and odor, and urinary tract infections?
	What impact would effective MHM products have in reducing transactional (or coerced) sex to obtain money for sanitary pads?
	How is girls’ psychosocial stress impacted by a lack of resources, guidance, and/or a non-supportive school environment for practicing MHM?
Research and strategies to strengthen advocacy and action	What MHM programs have successfully implemented activities and what are lessons learned? What added value can the Cochrane approach of systematic reviews and meta-analysis provide to aggregate and compare behaviors, impact, and cost-effectiveness of MHM interventions?
Girls’ empowerment and cultural norms	What contribution does improved MHM have toward improving girls’ lives and reducing gender inequity? How will girls’ self-efficacy in managing menstruation correlate to later decision-making about their bodies (i.e. age at first sex, sex negotiation, condom negotiation, and contraception use)?
	What are the experiences of girls who do not experience regular menstruation and how does this impact their life prospects (social isolation, marriage, etc.)? What effect do males have on girls’ ability to independently manage their menstruation, and engage in safe, healthy, productive, and meaningful activities?

MHM, menstrual hygiene management. WASH, water, sanitation, and hygiene.

## The need for standardized definitions and measures

While MHM has been defined, universal agreement is needed for outcome measures, including targets that indicate successful implementation and endpoints that require laboratory confirmation. Below are a number of outcome measures identified to be important that, to date, have either been examined with variation in their definition, precision, and reliability, or have been neglected (Table 2). Others listed have not yet been measured in association with MHM. Predictors are sub-grouped by main effect topic. Standard definitions have been developed by the Joint Monitoring Programme (Box 1); yet, a wide range of definitions of ‘good’ and ‘bad’ MHM have been used. In order for a robust MHM evidence base that allows for cross-study comparisons, standardized outcome endpoints will prove to be key.

**Table 2:** MHM outcome measures that need standardized definitions

Outcome measure	Outcome targets
MHM outcome measures	- Measuring girls’ MHM self-efficacy
	- Measuring girls’ ability to comfortably participate in class, self-confidence, and pain
	- Defining a quantifiable measure of ‘good menstrual hygiene’
School outcome measures	- Defining measures of improved engagement/concentration in school lessons
	- Defining how to measure ‘school absence’ as a quantifiable effect of poor menstruation while accounting for other factors to ensure accuracy
	- Defining dropout and reasons for dropout, enrolment and re-enrolment, grade repetition, gender-adjusted (parity) index, etc.
Health outcome measures	- Measuring menstrual hygiene practice impacts and MHM product effectiveness on urogenital tract infection - Measuring sexual risk among girls receiving menstrual products through coerced sex
	- Measuring violence associated MHM in the absence of WASH facilities
Economic outcome measures	- Measuring cost outcomes (cost-effectiveness, return on investments, costs of ‘case’ averted, estimate of full productivity due to completion of education, etc.)
Quality of life/well-being outcome measures	- Identifying appropriate measures of psychosocial health for girls, (e.g. mental distress, anxiety, and depression)
	- Defining ‘well-being’/‘quality of life’ indicators, that is, testing PEDSQL (7 and 23 items measuring physical, emotional, schooling, and social indices, respectively) and EuroQoL (EQ-5D-3L measuring mobility, self-care, usual activities, pain/discomfort, and anxiety/depression)
Program outcome measures	- Evaluating successful implementation of MHM-friendly WASH programs in schools - Monitoring the impact of guidelines and education materials - Identifying and validating MHM-related indicators to be included in multi-country national-level surveys to assess changes in trends and outcomes over time and correlation with other already measured indicators (facility access, type of materials used, etc.)

MHM, menstrual hygiene management; PEDSQL, Pediatric Quality of Life Inventory^TM^

## The need for standardized study methodologies

While the priority topics listed above may include methodological components, Table 3 specifically highlight study methodologies that need to be considered in future research designs A wider range of approaches also need to be employed to capture social and cultural factors that influence implementation. Certification of researchers in good clinical practice (i.e. global health training centre: https://globalhealthtrainingcentre.tghn.org/elearning/) and research in human participants (i.e. NIH training: https://phrp.nihtraining.com/users/login.php) are required to ensure ethical practice (across all studies, not specifically trials), and to encourage study rigor. These methodologies will require adherence to standardized guidelines and protocols, such as PRISMA (36), STROBE (37), and CONSORT (38). This will in turn strengthen the existing evidence base motivating increased investment and allowing for measurement of impact.

**Table 3:** Study methodologies to be considered in future research designs

Study design	Methodological components
Intervention trials	Large-scale and multi-site trials which follow CONSORT guidelines (http://www.consort-statement.org/consort-statement/checklist) (37) are needed to quantify the impact of MHM interventions in different cultures and settings (e.g. MHM products; WASH infrastructure; psychosocial information/education/communication materials; programs and program implementation).
Observational methods	This includes cohort, cross-sectional, and case-control studies, which should follow STROBE guidelines (http://www.strobe-statement.org/) (36). Longitudinal studies that are able to follow individuals over time are needed to assess the impact of MHM on health, educational, and economic outcomes over the life-course.
Participatory methods	In addition to typical qualitative methodologies (focus groups, in-depth and key informant interviews), participatory approaches are needed to increase engagement of girls, other beneficiaries, and stakeholders in planning MHM programs (i.e. participatory rural appraisal); these should follow COREQ guidelines. (http://intqhc.oxfordjournals.org/content/19/6/349)
Multi-disciplinary/mixed methods	Studies that generate health, social, and economic indicators, and examine inferences for program delivery.
Systematic reviews and meta-analysis	Reviews, following PRISMA guidelines (http://www.prisma-statement.org/) (35), are needed to confirm or refute findings from a range of prevalence and intervention studies across populations, and thus guide policy and best practice.
Operational research	Operational research is needed to understand how national governments are implementing MHM-friendly policies at scale, so that translation of policy into practice can be improved and these experiences can be leveraged elsewhere.
Natural experiments	Natural experiments provide an opportunity to evaluate the impact of national-level MHM-related policies or programs, like a new school-health policy that embraces MHM, a curricula change, or a nationwide campaign.

COREQ, consolidated criteria for reporting qualitative research; MHM, menstrual hygiene management.

Inadequate funding for MHM research has been a key factor contributing to many of the methodological issues within existing studies and the limited quantification of MHM in schools. Support is needed to strengthen the MHM study methodologies that have been applied as well as those that have not been undertaken to date. This is critical to ensure that future studies, including impact assessments and those that attempt to quantify outcomes, have adequate sample sizes, and that individual participants are followed up (as the unit of measurement), with loss to follow-up taken into account.

## The need for an MHM research consortia

To further facilitate the research agenda, a multi-disciplinary, cross-sectoral, and cross-cultural research consortia panel is required to foster high-quality research studies on priority topics to generate key findings relevant for global policy. An important component of the research agenda will be to provide a conduit for strengthening and supporting research quality through the sharing of methodologies and tools, and developing standard measurement criteria. The latter will allow comparison and pooling of findings across studies for meta-analyses, as well as contributing toward global north–south learning and capacity building. Envisaged functions of a research consortia are presented in Table 4.

**Table 4:** Function of the MHM Research Consortia

Function
Establish a network list of international MHM researchers Create a repository on completed, ongoing, and planned research studies Develop standard indicators for measuring impact Liaise and communicate with researchers, including on priority research topic listing, research findings, funding opportunities, and events Support research design with expert guidance, including statistical support for protocol development, that is, for sample size calculations on protocols, guidance on data management plans, statistical plans, and standard operating procedures Assist with research study registration, that is, Clinical Trials Network for trials, and quality assurance to ensure research reaches CONSORT standards Liaise with funders to update research priorities and develop new funding opportunities Set up a funding mechanism with agencies wishing to support high-quality research

MHM, menstrual hygiene management.

Such a consortia once *in situ* will offer an opportunity for funders to pool resources and establish a funding mechanism through the consortia peer-review research panel. Seed money would be needed in the first instance to develop the research network infrastructure, consortia terms of reference, objectives, milestones, timeframes, work packages, funding mechanisms and accountability system, communication strategies, and to initiate the design and provision of guidelines for researchers. Ultimately, we expect that this collaborative effort will facilitate the generation of local and global evidence-based findings that can be translated into policies and programs globally to promote MHM and improve adolescent girls’ dignity, health, well-being, and schooling, and enable girls to reach their full potential.

## Conclusions

This brief article represents a consensus by engaged experts from a range of disciplines, on the priority of MHM in schools’ research topics and methods that need to be explored in order to fill the gaps in the existing MHM evidence base. This collective effort aims to improve the quality and focus of research, enhance effectiveness of programming, strengthen global commitment, increase funding, facilitate sharing of expertise across disciplines to foster multi-disciplinary studies (including intervention research), and eliminate time and money spent on duplication of efforts in topic areas already well researched. We advocate for the creation of a global MHM research network through a research consortia that will help support the development of research guidelines, strengthen translation of evidence-based research into policy, and facilitate funding. Ultimately, while great advances have been made in the MHM research evidence base to date, there remain a number of important gaps in our collective knowledge. Filling these gaps will require new research studies that use an expanded range of methodologies, that enable the global community to better understand the magnitude of the problem surrounding MHM for adolescent girls (in and out of school), the impact of MHM interventions, and the costs incurred to implement them effectively. Without broadening the existing evidence-based knowledge, opportunities to improve an essential component of adolescent lives will remain limited.

## Paper context

There is a dearth of data measuring the effects of girls’ menses on health and well-being in low-and middle-income countries. This lack of research has been identified as one of the five top priorities for advancing good menstrual hygiene management (MHM) globally. This article highlights the existing gaps in MHM research and identifies opportunities to build hard evidence and improve the ability of governments, donors, and other agencies to appropriately target resources to support MHM.
